# Supplementary material for: Low Protein Expression of both ATRX and ZNRF3 as Novel Negative Prognostic Markers of Adult Adrenocortical Carcinoma
Source: Int J Mol Sci. 2021 Jan 27;22(3):1238. doi: 10.3390/ijms22031238 (PMC7866180; doi:10.3390/ijms22031238)
Supplement: Supplementary file 1 [file ijms-22-01238-s001.zip › ijms-1032047-sl-proofreading/Table S1_CoxResults.docx]

**Table S1.** Principal Cox regression results for OS

| Variables | Parameters | Coefficient | Standard error | HR | 95,0% CI | | *p* value |
| --- | --- | --- | --- | --- | --- | --- | --- |
|  |  |  |  |  | Inferior | Superior |  |
| ATRX expression | continuous | -0.198 | 0.105 | 0.821 | 0.668 | 1.008 | >0.05 |
|  | >1.5 | -0.651 | 0.331 | 0.521 | 0.273 | 0.997 | 0.049 |
| ZNRF3 expression | continuous | -0.141 | 0.071 | 0.869 | 0.757 | 0.998 | 0.046 |
|  | >2 | -0.818 | 0.336 | 0.441 | 0.229 | 0.852 | 0.015 |
| Age at diagnosis | continuous | 0.006 | 0.010 | 1.006 | 0.987 | 1.025 | 0.527 |
|  | >51years | 0.374 | 0.331 | 1.453 | 0.759 | 2.781 | >0.05 |
| Metastasis at diagnosis |  | 1.089 | 0.378 | 2.971 | 1.415 | 6.237 | 0.004 |
| Tumor weight | continuous | 0.001 | 0.000 | 1.001 | 1.000 | 1.001 | 0.001 |
|  | >55 grams | 3.665 | 1.714 | 39.052 | 1.357 | 1123.525 | 0.032 |
| Tumor size | continuous | 0.100 | 0.029 | 1.105 | 1.045 | 1.169 | <0.001 |
|  | >7 cm | 1.493 | 0.482 | 4.451 | 1.729 | 11.458 | 0.002 |
| Weiss Score | continuous | 0.331 | 0.090 | 1.392 | 1.167 | 1.661 | <0.001 |
|  | >4 | 1.396 | 0.386 | 4.040 | 1.895 | 8.611 | <0.001 |
| Presence of disease recurrence |  | 2.707 | 1.225 | 14.985 | 1.358 | 165.390 | 0.027 |
| ATRX and ZNRF3 expressions | ATRX>1.5 e ZNRF3≦2 | -0.678 | 0.447 | 0.507 | 0.211 | 1.218 | >0.05 |
|  | ATRX≦1.5 e ZNRF3>2 | -0.911 | 0.477 | 0.402 | 0.158 | 1.025 | >0.05 |
|  | ATRX>1.5 e ZNRF3>2 | -1.157 | 0.426 | 0.314 | 0.136 | 0.725 | 0.007 |
| Ki-67 and ATRX expressions | Ki-67>10% e ATRX≦1.5 | 0.253 | 0.453 | 1.288 | 0.530 | 3.128 | >0.05 |
|  | Ki-67≦10% e ATRX>1.5 | -0.835 | 0.551 | 0.434 | 0.147 | 1.276 | >0.05 |
|  | Ki-67>10% e ATRX>1.5 | 0.274 | 0.454 | 1.315 | 0.541 | 3.200 | >0.05 |
| Ki-67 and ZNRF3 expressions | Ki-67>10% e ZNRF3≦2 | 0.039 | 0.434 | 1.040 | 0.444 | 2.436 | >0.05 |
|  | Ki-67≦10% e ZNRF3>2 | -1.014 | 0.530 | 0.363 | 0.128 | 1.024 | >0.05 |
|  | Ki-67>10% e ZNRF3>2 | 0.249 | 0.509 | 1.282 | 0.473 | 3.477 | >0.05 |
| Ki-67 | >8 | 1.850 | 0.490 | 6.360 | 2.434 | 16.618 | <0.001 |
|  | ≧10% and <20% | 1.758 | 0.441 | 5.802 | 2.447 | 13.760 | <0.001 |
|  | ≧20% | 2.202 | 0.478 | 9.041 | 3.543 | 23.073 | <0.001 |
